# Supplementary material for: Factors Influencing Quality of Life in Breast Cancer Patients Six Months after the Completion of Chemotherapy
Source: Diseases. 2019 Feb 24;7(1):26. doi: 10.3390/diseases7010026 (PMC6473656; doi:10.3390/diseases7010026)
Supplement: Supplementary file 1 [file diseases-07-00026-s001.pdf]

Table 1. Association between age categories and clinical and demographic characteristics.

Characteristics	29-39 years old	40-49 years old	50-59 years old	Over 60 years old	χ^2(df), *p*
**Menopausal status**					46.06, p <.0005[2]
Pre-menopausal	8 (66.7%)[c,d]	6 (30%)[a]	0 (0%)[a]	0 (0%)	
Post-menopausal	0 (0%)[c]	3 (15%)[c]	11(84.6%)[a,b]	15 (93.8%)[a,b]	
Menopausal due to treatment	4 (33.3%)	11(55%)[d]	2 (15.4%)	1 (6.3%)[b]	
**Treatment Type**					2.71 (3), p = 46
Chemotherapy &surgery	6 (50%)	12 (60%)	4 (30.8%)	8 (50%)	
Chemotherapy &surgery&hormone therapy	6 (50%)	6 (40%)	9 (69.2%)	8 (50%)	
**Surgery**					1.53, p = 69[2]
Lumpectomy	9 (75%)	17 (85%)	10 (76.9%)	11 (68.8%)	
Mastectomy	3 (25%)	3 (15%)	3 (23.1%)	5 (31.3%)	
**Hematocrit**					.72, p = 92[2]
Low levels	3 (25%)	7 (35%)	5 (38.5%)	6 (37.5%)	
Normal levels	9 (75%)	13 (65%)	8 (61.5%)	10 (62.5%)	
**Hemoglobin**					3.87, p = .29[2]
Low levels	1 (8.3%)	7 (35%)	5 (38.5%)	6 (37.5%)	
Normal levels	11 (91.7%)	13 (65%)	8 (61.5%)	10 (62.5%)	
**White count**					.75, p = .90[2]
Low levels	4 (33.3%)	7 (35%)	5 (45.5%)	7 (43.8%)	
Normal levels	8 (66.6%)	13 (65%)	6 (54.5%)	9 (56.3%)	
**Duration of education**					.933, p = .12[2]

Primary education	1 (8.3%)	5 (25%)	2 (15.4%)	7 (46.7%)	
Secondary education (Middle + High School Graduates)	5 (41.7%)	5 (25%)	7 (53.8%)	6 (40%)	
Higher Education (University /Technological Institute or MA/PhD degrees)	6 (50%)	10 (50%)	4 (30.8%)	2 (13.3%)	
**Work status**					23.89, p = .001[2]
Blue collar	1 (8.3%)	5 (8.2%)	1 (7.7%)	0 (0%)	
White collar	6 (50%)[d]	7 (35%)	4 (30.8%)	0 (0%)[a]	
Housemakers	5 (41.7%)	7 (35%)	6 (46.2%)	8 (50%)	
Retired	0 (0%)[d]	1 (5%)[d]	2 (15.4%)	8 (50%)[a,b]	

[1]a: statistically different from 29-39y, b: statistically different from 40-49y, c: 50-59y, d: over 60y; [2]Fisher's exact test.

Table 2. Associations between treatment type and demographic and clinical characteristics.

Characteristics	Chemotherapy &surgery	Chemotherapy &surgery&hormone therapy	χ^2**(df),** ***p***
**Menopausal status**			1.99(2), p = .37
Pre-menopausal	9 (30%)	5 (16.1%)	
Post-menopausal	12 (40%)	17 (54.8%)	
Menopausal due to treatment	9 (30%)	9 (29%)	
**Surgery**			3.09 (1), p = .08
Lumpectomy	26 (55.3%)	4 (28.6%)	
Mastectomy	21 (44.7%)	10 (71.4%)	
**Hematocrit**			.51(1), p = .47
Low levels	9 (30%)	12 (38.7%)	
Normal levels	21 (70%)	19 (61.3%)	
**Hemoglobin**			.55(1), p =.46
Low levels	8 (26.7%)	11 (35.5%)	

Normal levels	22 (73.3%)	20 (64.5%)	
**White count**			.49(1), p =.49
Low levels	10 (34.5%)	13 (43.3%)	
Normal levels	19 (65.5%)	17 (56.7%)	
**Duration of education**			.11(2), p= 1
Primary education	7 (23.3%)	8 (26.7%)	
Secondary education (Middle + High School Graduates)	12 (40%)	11 (36.7%)	
Higher Education (University /Technological Institute or MA/PhD degrees)	11 (36.7%)	11 (36.7%)	
**Work status**			2.04(2), p = .57
Blue collar	5 (16.7%)	2 (6.5%)	
White collar	9 (30%)	8 (25.8%)	
Housemakers	11 (36.7%)	15 (48.4%)	
Retired	5 (16.7%)	6 (19.4%)	

Table 3. Associations between surgery type and demographic and clinical characteristics.

Characteristics	Lumpectomy	Mastectomy	χ^2(df), *p*
**Menopausal status**			6.23, p = .04[1]
a. Pre-menopausal	14 (29.8%)[c]	0 (0%)[lumbectomy,c]	
b. Post-menopausal	21 (44.7%)	8 (57.1%)	
c. Menopausal due to treatment	12 (25.5%)[a]	6 (42.9%)[a]	
**hematocrit**			.01, p =1[2]
Low levels	16 (34%)	5 (35.7%)	
Normal levels	31 (66%)	9 (64.3%)	
**Hemoglobin**			1.16(1), p .33
Low levels	13 (27.7%)	6 (42.9%)	

Normal levels	34 (72.3%)	8 (57.1%)	
**White count**			2.55(1), = .11
Low levels	15 (33.3%)	8 (57.1%)	
Normal levels	30 (66.7%)	6 (42.9%)	
**Duration of education**			2.09(2), p =.37
Primary education	10 (21.7%)	5 (33.3%)	
Secondary education (Middle + High School Graduates)	17 (37%)	6 (42.9%)	
Higher Education (University /Technological Institute or MA/PhD degrees)	19 (41.3%)	3 (21.4%)	
**Work status**			3.07, p = .36
Blue collar	7 (14.9%)	0 (0%)	
White collar	14 (29.8%)	3 (21.4%)	
Housemakers	18 (38.3%)	8 (57.1%)	
Retired	8 (17%)	3 (21.4%)	

[1]Fisher's exact test, [2]Pearson's chi-square exact test
